# Regulation of antilipopolysaccharide factors, ALF*Pm*3 and ALF*Pm*6, in *Penaeus monodon*

**DOI:** 10.1038/s41598-017-12137-5

**Published:** 2017-10-04

**Authors:** Pitchayanan Kamsaeng, Anchalee Tassanakajon, Kunlaya Somboonwiwat

**Affiliations:** Center of Excellence for Molecular Biology and Genomics of Shrimp, Department of Biochemistry, Faculty of Science, Chulalongkorn University, Phayathai Rd., Bangkok, 10330 Thailand

## Abstract

ALF*Pm*6, a member of antimicrobial peptide in the antilipopolysaccharide factor (ALF) family from *Penaeus monodon*, plays important roles in shrimp immunity against pathogens. However, its antimicrobial activity and underlying mechanism have not been reported. The synthetic cyclic ALF*Pm*6#29–52 peptide (cALF*Pm*6#29–52) corresponding to the ALF*Pm*6 LPS-binding domain can agglutinate and exhibited bacterial killing activity toward a Gram-negative bacterium, *Escherichia coli* 363 and Gram-positive bacteria, *Bacillus megaterium*, *Aerococcus viridans*, and *Micrococcus luteus*, with MIC values of 25–50 *μ*M. Specifically, ALF*Pm*6 and ALF*Pm*3, the most abundant ALF isoforms, are different in terms of gene expression patterns upon pathogen infections. Herein, the regulation of ALF*Pm*3 and ALF*Pm*6 gene expression was studied. The 5′-upstream and promoter sequences were identified and the putative transcription factor (TF)-binding sites were predicted. The narrow down assay indicated that the ALF*Pm*3 promoter and partial promoter of the ALF*Pm*6 active regions were located at nucleotide positions (−814/+302) and (−282/+85), respectively. Mutagenesis of selected TF-binding sites revealed that Rel/NF-*κ*B (−280/−270) of ALF*Pm*3 and C/EBP*β* (−88/−78) and Sp1 (−249/−238) sites of ALF*Pm*6 were the activator-binding sites. Knockdown of the *Pm*MyD88 and *Pm*Relish genes in *V*. *harveyi*-infected shrimp suggested that the ALF*Pm*3 gene was regulated by Toll and IMD pathways, while the ALF*Pm*6 gene was regulated by the Toll pathway.

## Introduction

Currently, white spot syndrome virus (WSSV) and bacteria of *Vibrio* species are considered major shrimp pathogens^1–3^. The major virulent strains of Vibrio in shrimp are *Vibrio harveyi*, *Vibrio alginolyticus*, and *Vibrio parahaemolyticus*, causing acute hepatopancreatic necrosis disease (AHPND)^4^. These virulent strains cause extreme losses of shrimp in hatcheries and grow-out farms, thus making the prevention and control of diseases a priority for shrimp production. To overcome these obstacles, knowledge regarding the innate immune system of shrimp is urgently required.

Antimicrobial peptides (AMPs) in the antilipopolysaccharide factor (ALF) family are important effectors in shrimp. ALFs are mostly highly cationic polypeptides of approximately 100 residues with a hydrophobic N-terminal region. ALFs contain a signal peptide at the N-terminus and a LPS-binding domain (LPS-BD). Two conserved cysteine residues between LPS-BD are involved in an intramolecular disulphide bridge. The differences in the LPS-BD sequences contribute to the binding capability of different ALF*Pm* isoforms to different microbial cell wall components^5^. The *P*. *monodon* ALF major isoform, ALF*Pm*3, exhibits a broad antimicrobial activity spectrum against filamentous fungi, Gram-positive and Gram-negative bacteria including the natural shrimp bacterial pathogens, *V*. *harveyi* causing vibriosis and *V*. *parahaemolyticus* AHPND causing early mortality syndrome^6,7^. The recombinant protein of ALF*Pm*3 (rALF*Pm*3) induce membrane permeabilization and cell lysis of bacterial cells^8^. The antiviral properties of rALF*Pm*3 against the major shrimp viral pathogen, WSSV, have been reported. ALF*Pm*3 inhibits WSSV propagation in crayfish hematopoietic cell culture and in shrimp^9^. It has recently been shown that ALF*Pm*3 performs its anti-WSSV activities by binding to the envelope proteins; WSSV189, WSSV395, and WSSV471; nucleocapsid proteins WSSV189; and tegument protein WSSV458^10,11^. Although ALF*Pm*3 is the major ALF isoform responsible for fighting pathogen infection, other ALF*Pm* isoforms such as the ALF*Pm*6 gene have been shown to be up-regulated in *P*. *monodon* haemocytes in response to yellow head virus infection^12^ and are important for the shrimp immune response to *V*. *harveyi*- and WSSV infections^13^. However, no reports have described ALF*Pm*6 activity. Moreover, knockdown of the ALF*Pm*3 gene causes rapid mortality, while gene silencing of ALF*Pm*6 has no effect on shrimp mortality but leads to a significant increase in cumulative mortality and a more rapid mortality rate following *V. harveyi* and WSSV infections, respectively^13^. Thus, the ALF*Pm*3 and ALF*Pm*6 genes might be controlled by different signalling pathways.

In *Drosophila*, the Toll and IMD pathways are clearly the two most important signaling pathways controlling antimicrobial peptide (AMP) genes^14–16^. The Toll pathway is mainly involved in defence against fungi, Gram-positive bacteria, and viruses^17–19^, while the IMD pathway plays key roles in controlling Gram-negative bacteria and virus infection. In shrimp, Toll-like receptors (TLRs) and Toll signaling proteins such as Sptzle, myeloid differentiation factor 88 (MyD88), and Dorsal also play regulatory roles on AMP genes upon pathogen infection. LvToll2 from *P*. *vannamei* can significantly activate the promoters of the NF-*κ*B pathway-controlled AMP genes, but not LvToll1 and LvToll3^20^. The up-regulation of Spätzle proteins from *Fenneropenaeus chinensis* (FcSpz) and *P*. *vannamei* (LvSpz1–3) during *Vibrio* species and WSSV infections affect the expression of certain AMPs^20,21^. The *Fc*MyD88 transcript from *F*. *chinensis* is up-regulated after bacterial infection^22^. Dual-luciferase reporter assays indicated that the Dorsal from *P*. *vannamei* (*Lv*Dorsal) could regulate the transcription of the shrimp penaeidin-4 gene^23^. IMD and their signalling proteins in the IMD pathway have been found in shrimp. *Lv*IMD and *Lv*Relish from *P*. *vannamei* are involved in penaeidin-4 gene regulation^24^. Moreover, LvIMD can activate the expression of *Lv*ALF-AA-K and *Lv*Relish transcripts in bacteria-challenged shrimp^25^. IMD from *F*. *chinensis* (*Fc*IMD) and *Procambarus clarkii* (*Pc*IMD) is involved in regulating the expression of several AMPs against Gram-negative bacteria in *F*. *chinensis* such as Crustin*Fc*1, Crustin*Fc*2, ALF*Fc*1, ALF*Fc*2 and Lys*Fc*1, and in *P*. *clarkii* such as Crustin*Pc*1, Crustin*Pc*3, ALF*Pc*6, ALF*Pc*8, and Lys*Pc*2. These results suggest that although the IMD distribution and expression patterns exhibit some differences, the IMD pathway may have a conserved function for AMP regulation in shrimp and crayfish immunity^26^. *Fc*Relish is necessary for the expression of penaeidin-5 against *Vibrio anguillarium* infection^27^. In *P*. *monodon*, it has been demonstrated that PmRelish can transcriptionally regulate penaeidin-5, but not ALF*Pm*3 and Crustin*Pm*1, in YHV-infected shrimp^28^.

Although the control of AMP and its promoter has been reported in several shrimp, the crucial transcription factor binding sites and promoter active region involved in ALF regulation have not been studied. Moreover, the comparative regulation of each isoform of ALF is unclear. In this study, the synthetic cyclic peptide (cALF*Pm*6#29–52) corresponding to ALF*Pm*6 LPS-BD was tested for antimicrobial activity and bacterial agglutination property. In addition, the difference in the expression levels of the ALF*Pm*3 and ALF*Pm*6 genes was explored by identifying of their promoter active regions and transcription factor binding sites that might play an important role in controlling gene expression. We also determined whether the Toll and IMD pathways were involved in regulating their gene expression.

## Results

### The synthetic peptide derived from ALF*Pm*6-LPS BD (cALF*Pm*6#29–52) exhibited antibacterial activity

It is known that the antimicrobial activity of ALF depends on the binding of ALF to pathogen cell wall components, which is mediated mainly via a positively charged cluster within the LPS-BD of ALF^29^. In our study, we attemped to produce the recombinant ALF*Pm*6 protein (rALF*Pm*6) in yeast *Pichia pastoris*. However, the rALF*Pm*6 protein was successfully produced but the purification could not be accomplished (data not shown). To access the activity of ALF*Pm*6, the synthetic cyclic peptide, cALF*Pm*6#29–52, corresponding to the LPS-BD of ALF*Pm*6 was examined for antimicrobial activity against various strains of Gram-negative bacteria and Gram-positive bacteria. The bactericidal activity of cALF*Pm*6#29–52 peptide was observed in comparison to the positive control. The cALF*Pm*6#29–52 peptide was able to kill a Gram-negative bacterium, *E*. *coli* 363, and some Gram-positive bacteria such as *B*. *megaterium*, *A*. *viridans*, and *M*. *luteus* with MBC values ranging from 25–50 *μ*M (Table 1).

**Table 1:** Range of bactericidal activity of the synthetic peptide ALF*Pm*6 against various strains of microorganisms using liquid growth inhibition assay.

Microorganism	MBC value* ﻿of﻿ cALF*Pm*6#29–52 (*μ*M)
Gram-positive bacteria	
*Aerococcus viridans*	25–50
*Bacillus megaterium*	25–50
*Micrococcus luteus*	25–50
*Staphylococus haemolyticus*	>100
Gram-negative bacteria	
*Enterobacter cloacae*	>100
*Erwinia carotovora*	>100
*Escherichia coli 363*	25–50
*Klebsiella pneumoniae*	>100
*Vibrio haemolyticus (AHPND)﻿﻿﻿﻿﻿*	>100
*Non*-*virulent Vibrio haemolyticus*	>100
*Vibrio harveyi*	>100

*MBC value is expressed as the interval *a*-*b*, where *a* is the highest concentration tested at which microorganisms are growing and *b* the lowest concentration tested to kill particular bacterium.

### The synthetic peptide cALF*Pm*6#29–52 induced bacterial agglutination *in vitro*

**Figure 1:**
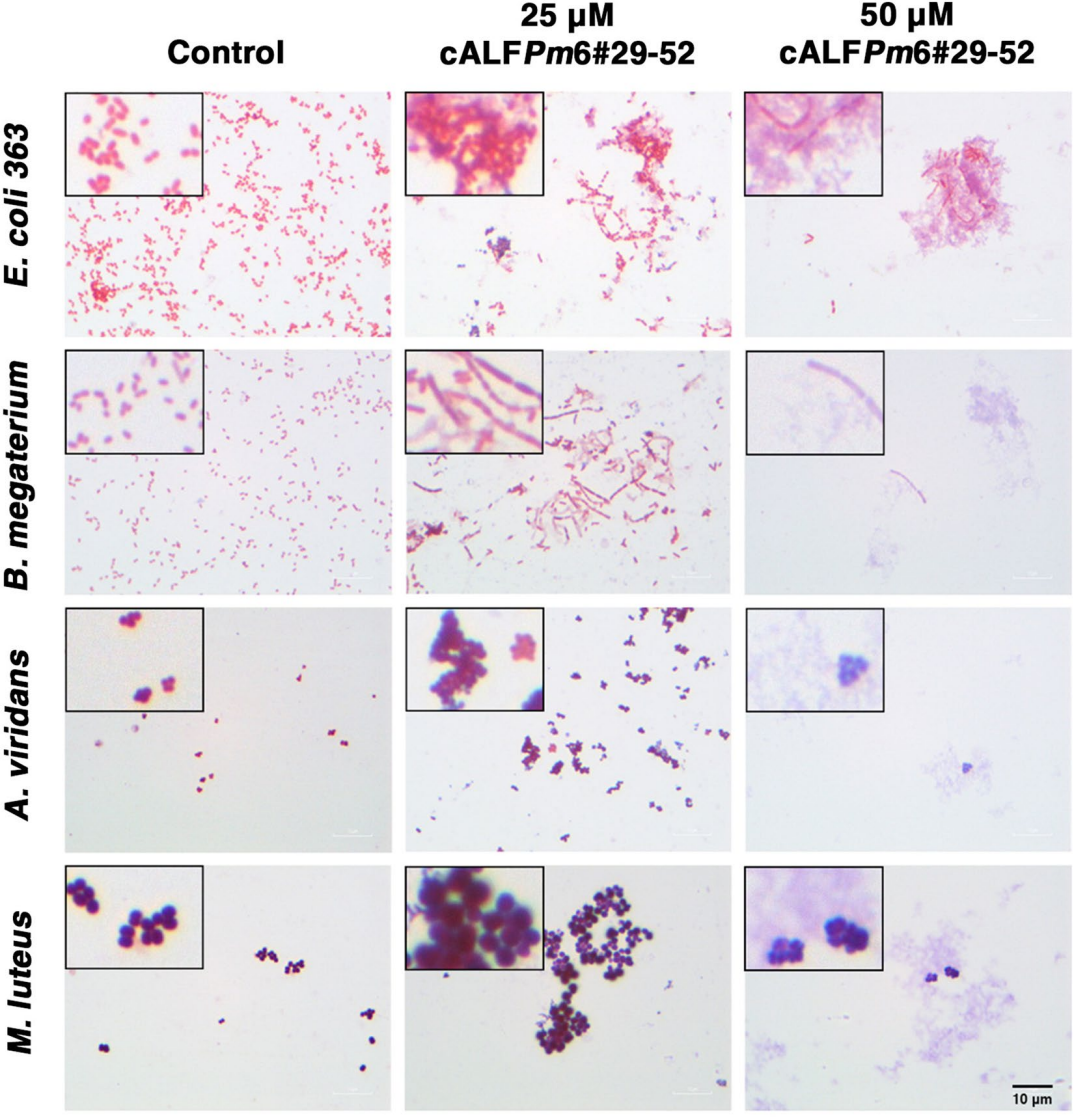
Bacterial agglutination mediated by the synthetic peptide cALF*Pm*6#29–52. *Escherichia coli* 363, *Bacillus megaterium*, *Aerococcus viridans*, or *Micrococcus luteus* was incubated overnight with the synthetic cALF*Pm*6#29–52 at a 25 and 50 *μ*M final concentration. Agglutination was observed a 100X magnification using a light microscope (Olympus CX31). Images were obtained using a Nikon DS-Fi1. The scale bar is 10 *μ*m.

There is evidence showing that some antimicrobial proteins and peptides can agglutinate bacteria cells. In this study, cALF*Pm*6#29–52 was tested for bacterial agglutination properties. We showed herein that the synthetic cALF*Pm*6#29–52 induced bacterial agglutination of *E*. *coli* 363, *B*. *megaterium*, *A*. *viridans*, and *M*. *luteus* at a 25 *μ*M final concentration. At 50 *μ*M, cALF*Pm*6#29–52 could completely inhibit the bacterial growth, and only cell debris could be detected under microscopic observation (Fig. 1). These results suggested that cALF*Pm*6#29–52 not only agglutinates bacteria but also exhibits bactericidal activity against sensitive strains in a dose-dependent manner.

### Determination of regulatory elements of ALF*Pm*3 and ALF*Pm*6 genes

The expression profiles of ALF*Pm*3 and ALF*Pm*6 genes upon pathogen infection of shrimp are different^13^. To better understand how both genes are regulated, the sequences and activity of their promoters were identified and characterized.

**Figure 2:**
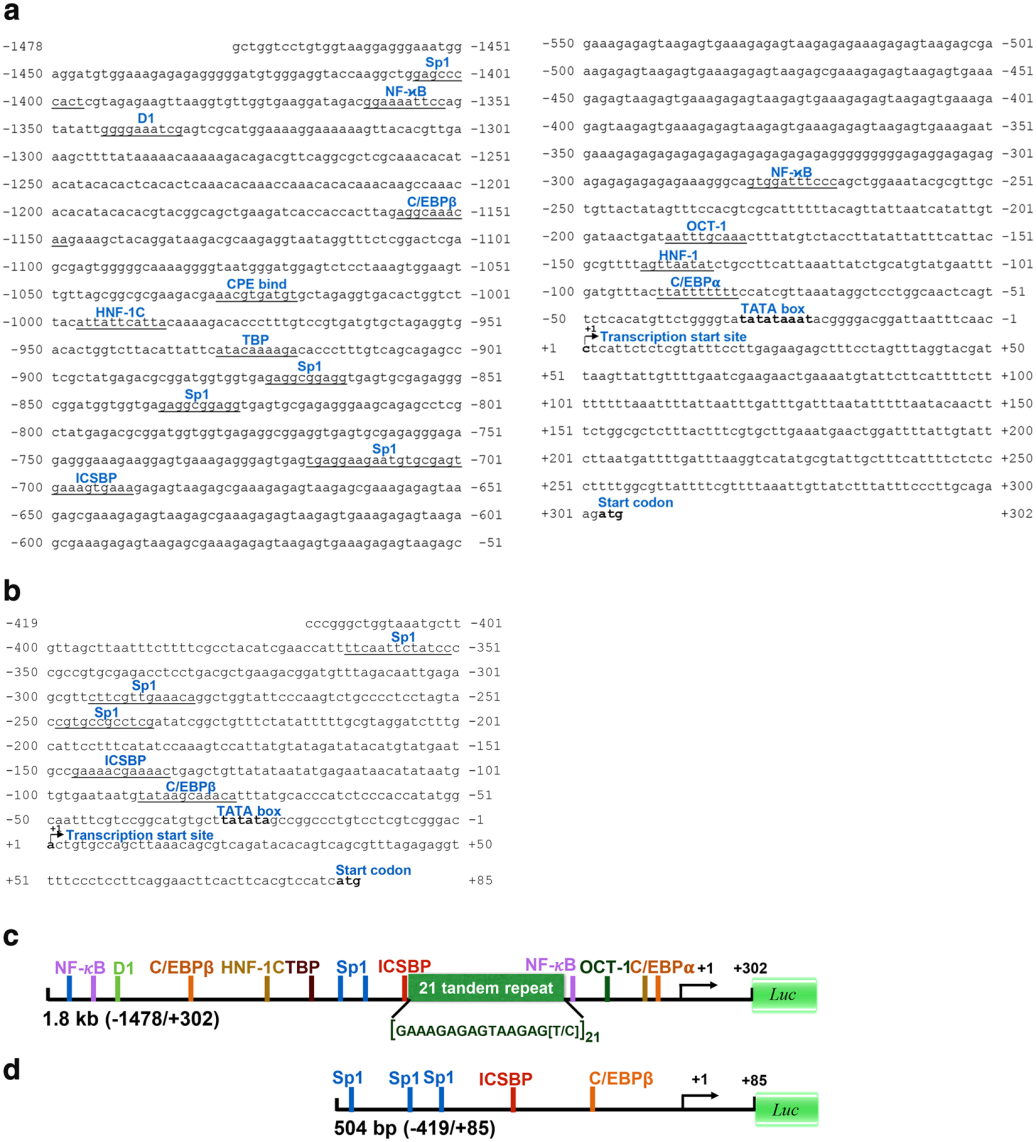
Upstream region and putative transcription factor binding sites of ALF*Pm* gene. The genomic sequence of the 5′-upstream region of the ALF*Pm*3 (**a**) and ALF*Pm*6 (**b**) genes. The putative cis-regulatory elements in the 5′-flanking promoter region are underlined. The transcription start site (+1) is indicated by the arrow labelled +1. The start codon and TATA box are also indicated, and their sequences are bolded. Schematic diagrams of the ALF*Pm*3 (**c**) and ALF*Pm*6 (**d**) promoter constructs used to delineate the TF-binding sites are shown.

The 5′-upstream genomic sequences of ALF*Pm*3, with a length of 1,478 bp, and ALF*Pm*6, with a length of 419 bp, were identified by genome walking. The positions of the transcription start site (+1) of the ALF*Pm*3 and ALF*Pm*6 genes were located at nucleotide positions −302 and −85, respectively, 5′-upstream from the start codon (ATG). The TATA box of ALF*Pm*3 and ALF*Pm*6 genes were located from −31 to −23 and from −29 to −24 5′-upstream from the transcription start site (+1), respectively (Fig. 2).

For the ALF*Pm*3 gene, several TF-binding sites such as specificity protein 1 (Sp1), nuclear factor NF-*κ*B binding sites, CCAAT-enhancer-binding proteins beta (C/EBP*β*), CCAAT-enhancer-binding proteins alpha (C/EBP*α*), octamer-1 (OCT-1), hepatocyte nuclear factors-1 (HNF-1), hepatocyte nuclear factors-1C (HNF-1C), D1, cytoplasmic polyadenylation element binding protein (CPE bind) and interferon consensus sequence-binding protein (ICSBP), were predicted (Fig. 2a,c). For the ALF*Pm*6 gene, TF-binding sites including Sp1, C/EBP*β*, and ICSBP, were predicted (Fig. 2b,d).

### Identification of transcription factor binding sites that regulate ALF*Pm*3 and ALF*Pm*6 gene expression

**Figure 3:**
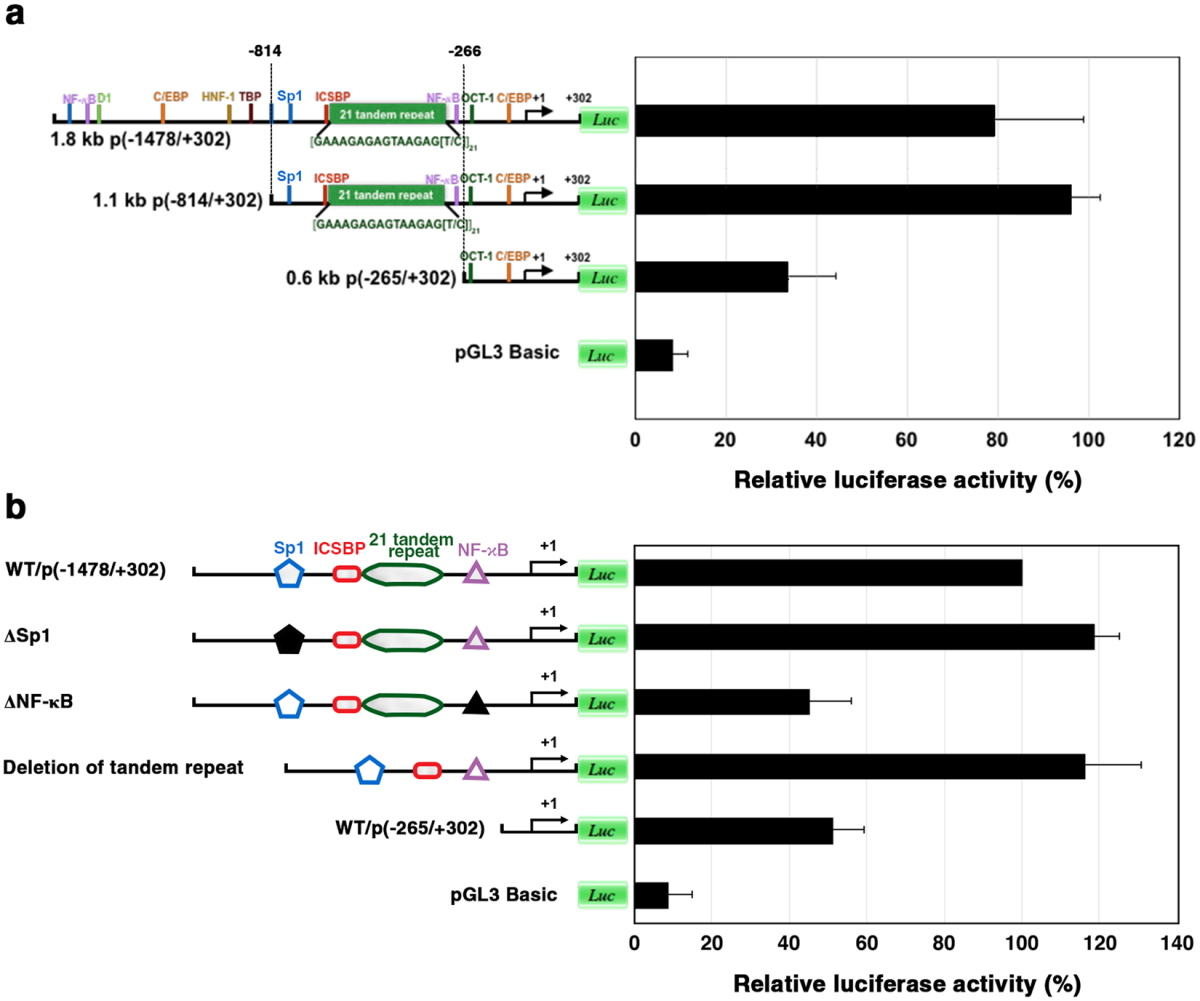
Functional analysis of the ALF*Pm*3 promoter through serial deletion and site-directed mutagenesis experiments. (**a**) Functional mapping of the ALF*Pm*3 promoter deletion. For promoter region −1478 to −265, the reporter vector containing ALF*Pm*3 promoter fragments of (−1478/+302), (−814/+302) and (−265/+302) was constructed. (**b**) Site-directed mutagenesis indicated by closed symbols of the transcription factor binding site Sp1 and NF-*κ*B on the ALF*Pm*3 promoter active region (−814/−266) and deletion of the 21 units of [GAAAGAGAGTAAGAG(T/C)] tandem repeat at position −693 to −358 from the ALF*Pm*3 promoter region. Relative luciferase activity (%) of each construct is shown. Data represent the means ± standard deviations from three independent experiments. The error bars denote standard deviations.

The regulatory regions on the ALF*Pm*3 and ALF*Pm*6 promoter sequences were identified by the narrow down assay. Following the ALF*Pm*3 promoter activity assay, the promoter active region (−814/+302) was identified. Narrow down of the promoter fragment to −265/+302 showed the lowest activity (Fig. 3a), suggesting that the promoter sequence at position −814 to −266 might contain the activator-binding site. The DNA sequence at position −814 to −266 of the ALF*Pm*3 promoter active region contained many TF-binding sites such as Sp1 at position −719 to −701, ICSBP at position −700 to −691, 21 units of GAAAGAGAGTAAGAG[T/C] tandem repeats at position −693 to −358 and NF-*κ*B at position −280 to −270.

To further investigate the element responsible for ALF*Pm*3 gene regulation, a deletion assay for 21 units of GAAAGAGAGTAAGAG[T/C] tandem repeats and site-directed mutagenesis at the transcription factor binding sites, Sp1 and NF-*κ*B were performed.

The 21 units of GAAAGAGAGTAAGAG[T/C] tandem repeat-deleted construct, del(−693/−358)/p(−1478/+302), was cloned and assayed for promoter activity. The promoter activity of del(−693/−358)/p(−1478/+302) was not significantly different when compared with the control p(−1478/+302) (Fig. 3b). These results suggested that the 21 units of the GAAAGAGAGTAAGAG[T/C] tandem repeat and ICSBP transcription factor binding site were not involved in ALF*Pm*3 gene regulation.

Site-directed mutagenesis was performed to determine whether Sp1 and Rel/NF-*κ*B transcription factor binding sites are involved in regulating the ALF*Pm*3 gene. The −814/−266 ALF*Pm*3 promoter region, was mutated at the transcription factor binding sites of Sp1 (−719/−701) and NF-*κ*B (−280/−270) by rolling PCR using p(−1478/+302) as a template. The mutated plasmids, ΔSp1/p(−1478/+302) and ΔNF-*κ*B/p(−1478/+302), were assayed for the promoter activity in comparison with the wild type plasmid p(−1478/+302). No significant change in promoter activity was observed with ΔSp1/p(−1478/+302). In constrast, mutation at the NF-*κ*B binding site caused a decrease in promoter activity by 46.6% compared with the wild type plasmid (Fig. 3b), suggesting that the NF-*κ*B binding site is an activator binding site on the ALF*Pm*3 promoter.

**Figure 4:**
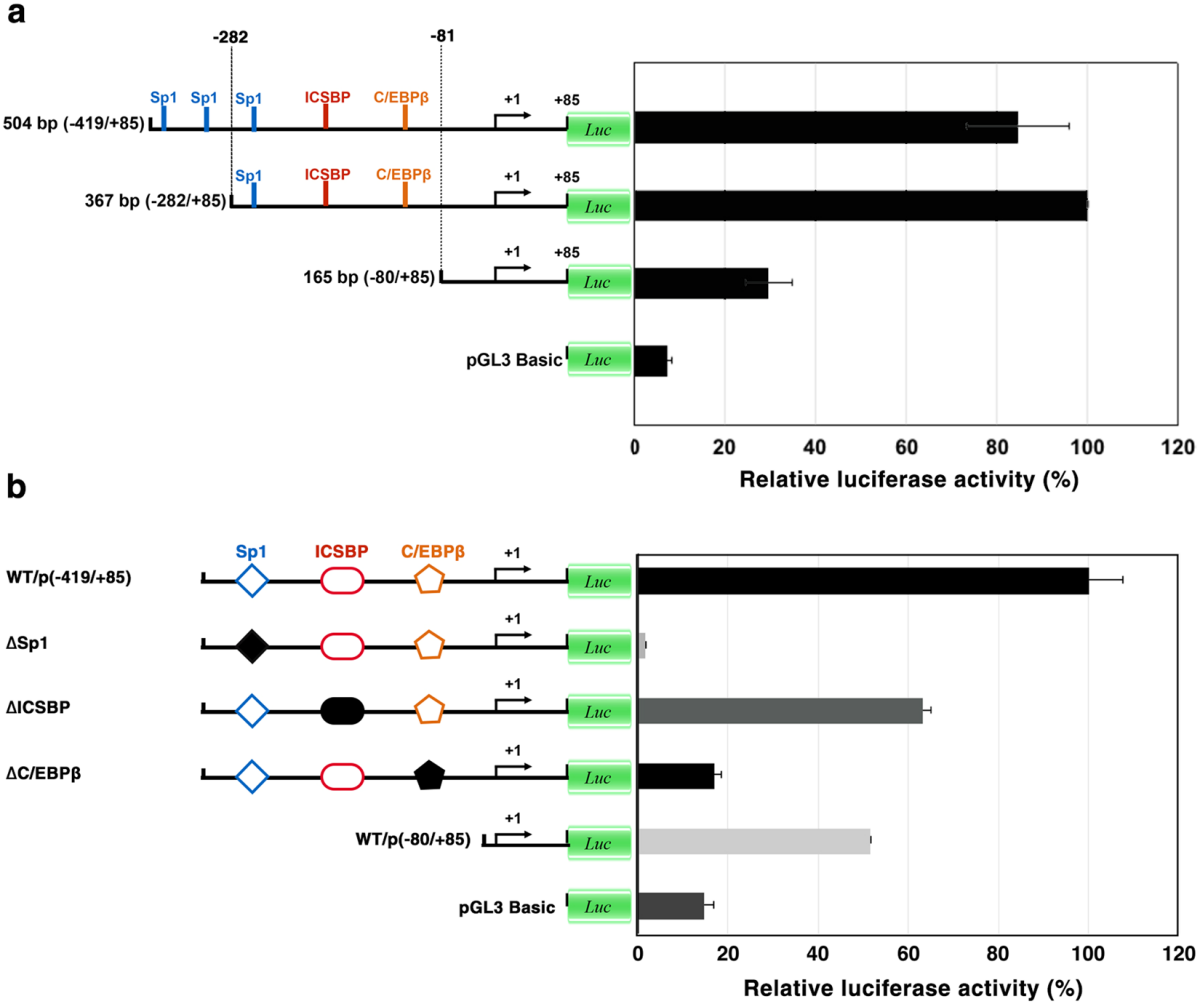
Functional analysis of the partial promoter of ALF*Pm*6 through serial deletion and site-directed mutagenesis experiments. (**a**) Functional mapping of the ALF*Pm*6 promoter sequence deletion from position −419 to −80. The reporter vector containing ALF*Pm*6 promoter fragments (−419/+85), (−282/+85) and (−80/+85) was constructed. (**b**) Site-directed mutagenesis indicated by closed symbols of transcription factor binding site Sp1, ICSBP and C/EBP*β* on the ALF*Pm*6 promoter active region (−282/−81). ﻿Relative luciferase activity (%) of each construct is ﻿shown﻿. Data represent the means ± standard deviations from three independent experiments. The error bars denote the standard deviations.

The promoter activity of each ALF*Pm*6 promoter fragment was determined. Compared to the control, the promoter fragment −282/+85 had the highest promoter activity whereas the promoter fragment −80/+85 showed the lowest promoter activity (Fig. 4a). The ALF*Pm*6 promoter active region (−282/+85) might have the activator-binding site at position −282 to −81. It contained many transcription factor binding sites such as Sp1 at position −249 to −238, ICSBP at position −146 to −135 and C/EBP*β* at position −88 to −80.

For the −282/−81 ALF*Pm*6 promoter region, the mutated reporter plasmids at the transcription factor binding sites of Sp1 (−249/−238), ICSBP (−146/−135) and C/EBP*β* (−88/−80) were constructed by rolling PCR using p(−419/+85) as a template. In comparison to the wild type plasmid p(−419/+85), the mutated plasmids, ΔICSBP/p(−419/+85), ΔC/EBP*β*/p(−419/+85), and ΔSp1/p(−419/+85), showed an approximately 36.8%, 83.1%, and 98.4% decrease in promoter activity, respectively (Fig. 4b). Our results indicated that these binding regions are involved in ALF*Pm*6 gene regulation and might have different binding affinities for transcription factors that resulted in different activation strengths of ALF*Pm*6 gene expression.

### Expression of the ALF*Pm*3 and ALF*Pm*6 genes is regulated by Toll and IMD pathways

The above results suggest that ALF*Pm*3 and ALF*Pm*6 genes are regulated by different transcription factors. However, the pathways that control their expression are still unclear. In this study, MyD88 and Relish, which are the representative adapter proteins of the Toll and IMD pathways, respectively, were silenced in *V*. *harveyi*-challenged shrimp in order to determine which signalling pathway plays a role in regulating ALF*Pm*3 and ALF*Pm*6 gene expression.

**Figure 5:**
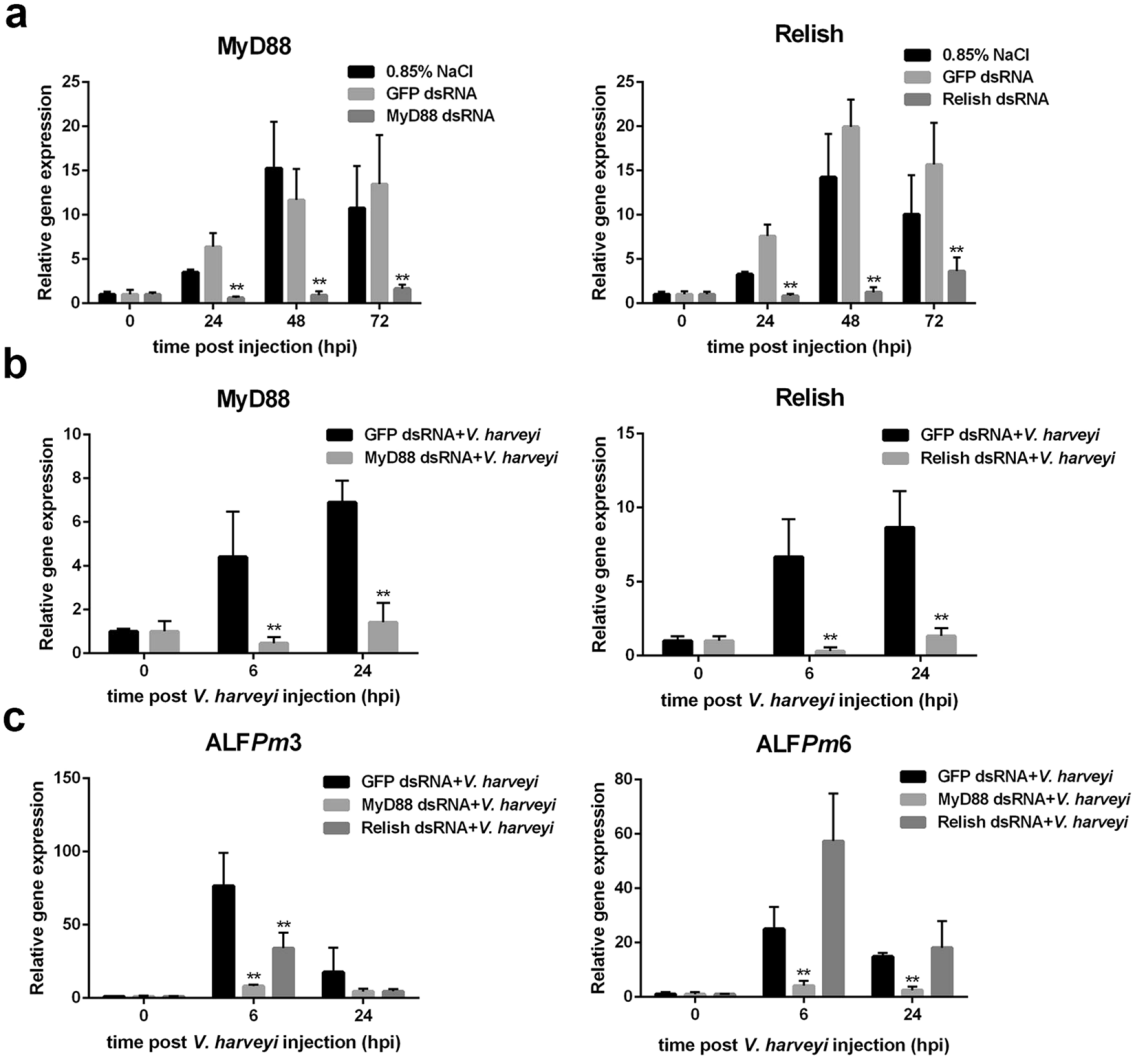
The effect of MyD88 and Relish gene knockdown on ALF*Pm*3 and ALF*Pm*6 transcripts levels upon *V*. *harveyi* infection. (**a**) Efficiency of MyD88 and Relish gene silencing mediated by dsRNA in shrimp. Control groups were injected with 0.85% NaCl or GFP dsRNA. The experimental groups were shrimp injected with 10 *μ*g/g shrimp MyD88 dsRNA and Relish dsRNA. At 0, 24, 48 and 72 h post-dsRNA injection, haemolymph was collected from 36 individual shrimp and randomly divided into 3 pools (n = 3 in each pool). The transcription levels of MyD88 or Relish were analysed using qRT-PCR. Elongation factor-1*α* (EF1*α*) was used as an internal control. (**b**) MyD88 and Relish gene silencing in *V. harveyi*-infected shrimps. The transcriptional level of MyD88 and ﻿Re﻿lish﻿ genes in the haemolymph o﻿f MyD88 dsRNA ﻿and Relish dsRNA-﻿﻿challenged shrim﻿p﻿, respectively, at 0, 6 and 24 h post-*V*. *harveyi* infection was determined. The control was shrimp injected with GFP dsRNA. EF1*α* was used as the internal control gene. (**c**) Silencing of the MyD88 and Relish genes affected the expression of the ALF*Pm*3 and ALF*Pm*6 genes. The expression of the ALF*Pm*3 and ALF*Pm*6 genes in the MyD88 and Relish gene-silenced shrimp upon *V*. *harveyi* challenge was determined by qRT-PCR. The control was shrimp injected with GFP dsRNA. The ALF*Pm*3 and ALF*Pm*6 gene expression levels were normalized to EF1*α*. The expression of the ALF*Pm*3 and ALF*Pm*6 genes in the *V*. *harveyi*-infected group at 6 and 24 hpi was normalized to that of the control group at 0 hpi. The graph shows the means ± standard deviations of 3 independent sets, **P < 0.01.

To test the efficiency of MyD88 dsRNA or Relish dsRNA in silencing the gene expression in shrimp, the expression levels of the MyD88 and Relish transcripts, respectively, were determined after injection of 10 *μ*g/g shrimp of the specific dsRNA into shrimp at 0, 24, 48 and 72 h by quantitative real-time PCR (qRT-PCR) using EF1*α* as an internal control. Normal saline- and GFP dsRNA-injected shrimp were used as negative controls. The result showed that MyD88 gene expression was suppressed at 0–72 h post-MyD88 dsRNA injection. In contrast, expression of the Relish gene was suppressed at 0–48 h post-Relish dsRNA injection and slightly recovered at 72 h post dsRNA-Relish injection (Fig. 5a).

The transcript levels of ALF*Pm*3 and ALF*Pm*6 transcripts in haemocytes of *V*. *harveyi*-challenged shrimp in the presence of MyD88 and Relish gene knockdown were determined by ﻿qRT-PCR. Before ALF*Pm*3 and ALF*Pm*6 expression analysis, the suppression of MyD88 and Relish transcripts in *V*. *harveyi*-infected shrimp was confirmed by qRT-PCR. Note that in *V*. *harveyi*-infected shrimp, GFP dsRNA had no effect on Relish and MyD88 mRNA expression levels (Fig. 5b). After challenging MyD88-silenced shrimp with *V*. *harveyi*, the expression of ALF*Pm*3 transcript sharply decreased at 6 and 24 hpi by 89% and 74.7%, respectively, when normalized to that of dsGFP knockdown shrimp. Knockdown of the Relish gene also resulted in a gradual decrease in ALF*Pm*3 gene expression from 6 hpi (53.9%) to 24 hpi (74.3%) (Fig. 5c). It is likely that ALF*Pm*3 was regulated by both Toll and IMD pathways. ALF*Pm*6 gene expression in MyD88 knockdown shrimp was greatly decreased at 6 hpi (82.9%) and 24 hpi (80.1%) when compared to that of dsGFP knockdown shrimp. In contrast, ALF*Pm*6 gene expression increased by 34.3% at 6 hpi, and no difference was observed at 24 hpi between Relish knockdown shrimp and dsGFP-challenged shrimp (Fig. 5c). Our results suggested that Toll rather than the IMD pathway might play a greater role in the transcriptional regulation of the ALF*Pm*6 gene.

## Discussion

ALF comprises a group of antimicrobial peptides that have been identified and characterized in shrimp and other crustaceans. Six isoforms of ALF have been discovered in *P*. *monodon*. ALF*Pm*6 is the most recent and the second most abundant isoform identified in the *P*. *monodon* EST database (http://pmonodon.biotec.or.th)^30^. A previous report has indicated that the ALF*Pm*6 gene is up-regulated upon YHV infection^12^. Moreover, silencing of the ALF*Pm*6 gene revealed that it is essential for shrimp survival and plays protective roles in shrimp against *V. harveyi* and WSSV infections^13^. These findings suggest a potential role for ALF*Pm*6 in the shrimp immunity. However, no reports have characterized ALF*Pm*6. A previous study showed that the synthetic peptide of ALF LPS-BD could interact with LPS *in vitro* ^31^. Additionally, the synthetic cycled loop peptide designed based on LPS-BD of *P*. *monodon*, *Scylla paramamosain*, and *Scylla serrata* showed inhibition effects on the growth of Gram-negative bacteria, such as *V*. *harveyi*, *Pseudomonas aeruginosa* and *E*. *coli*, and a Gram-positive bacterium, *M*. *luteus* ^32–34^. In this research, the antimicrobial activity of the synthetic cyclic LPS-BD peptide, cALFPm6#29–52, exhibited antimicrobial activity against a Gram-negative bacterium, *E*. *coli* 363 with MBC ranging from 25 to 50 *μ*M and Gram-positive bacteria, including *B*. *megaterium*, *M*. *luteus* and *A*. *viridan*, with MBC ranging from 25 to 50 *μ*M. The cALF*Pm*6#29–52, thus, showed less antimicrobial activity than the synthetic peptide of ALF*Pm*3#35–51, which could act against a broader range of Gram-negative and Gram-positive bacteria^6^. It is noteworthy that the amino acid composition and the total numbers of positively charged amino acid residues (Arg and Lys) of LPS-BD of ALF*Pm*3 and ALF*Pm*6 were different. The LPS-BD of ALF*Pm*3 had 4 Lys and 2 Arg, while ALF*Pm*6 contained 3 Arg and 2 Lys with a net charge of LPS-BD of 6 and 5, respectively. Although the pI values of ALF*Pm*3 (pI = 9.81) and ALF*Pm*6 (pI = 9.96) affect their antimicrobial activities, it could not be responsible for the different antimicrobial activities in each isoform.

Generally, AMPs exhibit antimicrobial activity via several mechanisms. ALF*Pm*3 exhibits the antibacterial activity by binding to bacterial cell wall components and permeabilizing the bacterial membrane to cause a loss of membrane integrity and cell lysis^8^. Several AMPs not only exhibit antibacterial activity but also the agglutination activity. Bacterial agglutination activity has been reported for rcrustin*Pm*1 and rcrustin*Pm*7, a family of antimicrobial peptides from *P*. *monodon* ^35^. In the mud crab, *S. serrata*, haemocyanin can bind to bacterial cells and mediated agglutination through the recognition of bacterial OmpA and Ompx proteins^36^. rEs-DWD1 from the Chinese mitten crab (*Eriocheir sinensis*) causes significant aggregation of *B*. *subtilis* and *P*. *pastoris* ^22^. The synthetic peptide, GL13NH2, derived from the parotid secretory protein, agglutinates both Gram-negative and Gram-positive bacteria, including the oral pathogen *Aggregatibacter actinomycetemcomitans* and the oral commensal *Streptococcus gordonii*, but it does not showed bactericidal activity. Increasing the calculated net charge of GL13NH2 peptide from +1 to +5 resulted in the loss ability of bacterial agglutination but a gain in bactericidal activity against against *Pseudomonas aeruginosa*, *S*. *gordonii* and *E*. *coli* ^37^. In the case of cALF*Pm6*#29–52, it could agglutinate *E*. *coli* 363, *B*. *megaterium*, *M*. *luteus* and *A*. *viridian* and kill those bacteria. Taken together, we can conclude that different isoforms of ALFs have different abilities to kill bacteria via different mechanisms.

In addition to the difference in activity, previous studies have also shown that different ALF isoforms are expressed at different levels in response to microbial infection, which could be due to the disparity in gene regulation. Herein, the promoter regions of the ALF*Pm*3 and ALF*Pm*6 genes were characterized. Previously, approximately 600 bp of ALF*Pm*3 5′-upstream sequences from the transcription start site were obtained by the genome walking technique. The putative promoter of ALF*Pm*3 was identified at position −29 of the 5′-upstream sequences. Several transcription factor (TF)-binding sites, including octamer (OCT-1), GATA, CCAAT box and GAAA motifs, were predicted in the 5′-upstream sequences^38^. However, the promoter activity has not been tested. In ALF*Fc* from *F*. *chinensis*, immune-related TF-binding sites such as one AP4, one NF-*κ*B, one Sp-1, two GAAA, three OCT-1, and three GATA, were identified in the region from −702 to +1. Analysis of the ALF*Fc* promoter activity in insect Sf9 cell lines showed that the putative promoter region from −702 to +33 was induced by lipopolysaccharide or (1,3)-*β*-D-glucan, but the shorter promoter sequence pALF-318 (from −318 to +33) could only be induced only by (1,3)-*β*-D-glucan^39^. However, the important TFs controlling ALFs transcription remain uncharacterized.

In this study, the promoter active regions with designated the regulatory regions involved in regulating ALF*Pm*3 and ALF*Pm*6 gene expression were identified using the narrow down technique. From our analysis, we concluded that the ALF*Pm*3 promoter region at the position (−814/−266) contained the activator-binding site. The genomic DNA sequence of the ALF*Pm*3 promoter at position −814 to −266 contained many TF-binding sites such as Sp1, ICSBP, and NF-*κ*B. Tandem repeats of DNA are prevalent and hypervariable in higher eukaryotic genomes. TR’s of DNA represent a section of the genome that may be highly evolvable, as repeat numbers can change with frequencies 100–10000 higher than point mutations^40^. The numbers of tandem repeats present in coding regions, promoters, and introns have demonstrated functional roles in modulating protein activity and gene expression and a correlation with disease^41–43^. However, the functional role of intergenic tandem repeats that are found far away from coding regions is unclear. A deletion assay for 21 units of GAAAGAGAGTAAGAG[T/C] tandem repeats and ICSBP suggested that this tandem repeat and ICSBP were not involved in ALF*Pm*3 gene regulation. In contrast, Sp1 and NF-*κ*B might be repressor- and activator-binding sites, respectively, on the ALF*Pm*3 promoter. Rel/NF-*κ*B family genes play a central role in the transcription of innate immune effectors^44^. The NF-*κ*B binding sites were found in different antimicrobial peptide (AMP) genes, including penaeidin, crustin and ALF*Pm*2^38,45,46^. In this study, the NF-*κ*B binding sites of ALF*Pm*3 were also found at nucleotide position −280 to −270 from the transcription start sites. The NF-*κ*B family genes, Relish and Dorsal, could regulate the transcription of shrimp AMP genes such as Pen4, Pen5, crustin, and ALF^20,23,27^. The transcription of other antimicrobial peptides in shrimp including crustin and ALF, was also regulated by Relish^47^. In this study, using site-directed mutagenesis, the NF-*κ*B binding site at position (−280/−270) was identified as the activator-binding site that regulated the ALF*Pm*3 gene regulation.

Compared to the ALF*Pm*3 promoter, the ALF*Pm*6 promoter fragment obtained is relatively short and could probably only part of its regulatory region, although the important regulatory elements could be identified. After deletion of the promoter region at position (−282/−81), the promoter activity was decreased. Based on this result, we concluded that the ALF*Pm*6 promoter region at the position (−282/−81) contained the activator-binding site. The predicted transcription factor-binding sites identified here were Sp1, C/EBP*β* and ICSBP, which were shown to be involved in the positive regulation of ALF*Pm*6 gene by site-directed mutagenesis. Previously, the C/EBP*β* binding site was identified as one member of the transcription factor binding motif of the crustin*Pm*7 gene^45^. C/EBP*β* is a member of a family of transcription factors involved in important physiological processes, such as cellular proliferation and differentiation, regulation of energy homeostasis, inflammation, and haematopoiesis. Previously, LPS-induced C/EBP*β* was shown to specifically bind to the C/EBP response element in the SerpinB2 proximal promoter in mice, and loss of C/EBP*β* abrogates constitutive SerpinB2 gene transcription and the response to LPS^48^. C/EBP*β* has been reported to physically interact with AP-1 and NF-*κ*B to promote gene expression of inflammatory mediators^49^. Sp1 is a ubiquitous nuclear factor that plays a key role in maintaining the basal transcription of house-keeping genes^50^. According to previous reports, Sp1 and NF-*κ*B are key transcription factors in the regulation of CD40 gene. Sp1 is a key transcription factor controlling the basal expression of CD40. In LPS-stimulated cells, the transcription factor NF-*κ*B up-regulates CD40 expression. In contrast, Sp1 is phosphorylated and its DNA binding activity is reduced after LPS stimulation^51^. Therefore, it can be hypothesized that Sp1 is also involved in ALF*Pm*3 and ALF*Pm*6 gene regulation. However, further investigations are needed. In summary, our studies showed that NF-*κ*B at nucleotide position (−280/−270), C/EBP*β* at nucleotide position (−88/−80) and Sp1 at nucleotide position (−249/−238) in the ALF*Pm*3 and ALF*Pm*6 promoter active region, respectively, are necessary for positive regulation.

Toll and IMD pathways are the important NF-*κ*B signalling pathways controlling the expression of antimicrobial peptide genes^14–16^. Previously, the expression profiles of Toll and IMD in bacterial-challenged shrimp were detected and found to be up-regulated at an early stage in haemocytes and hepatopancreas after challenge with *M. lysodeikticu* and *V. anguillarum*. Furthermore, ALF expression was significantly increased in response to *V*. *anguillarum* and *M*. *lysodeikticu* challenge^52^. In this study, silencing of the adaptor protein, MyD88, and the transcription factor, Relish, which are key molecules in the Toll and IMD pathways, respectively, was performed to investigate the pathway responsible for controlling the gene expression of each ALF. Instead of down-regulation of the ALF*Pm*6 gene, up-regulation of the ALF*Pm*6 gene was observed at 6 and 24 hpi in Relish knockdown shrimp. These findings suggested that the IMD pathway was not involved in the regulation of ALF*Pm*6 gene expression. However, the positive effect of Relish knockdown on ALF*Pm*6 gene expression remains unclear and necessitates further investigation. It is noteworthy that the transcription levels of ALF*Pm*3 in MyD88- and Relish-silenced shrimp were down-regulated at 6 h post-*V*. *harveyi* infection. Therefore, ALF*Pm*3 gene expression was probably regulated by both Toll and IMD pathways. A recent study showed that the expression of ALF*Pm*3 was not regulated by *Pm*Relish upon yellow-head virus (YHV) infection. In *V*. *harveyi*-infected shrimp, it has been reported that the expression of ALF*Pm*3 relies not only on the *Pm*Relish-mediated pathway but also on another un-identified signalling pathway^53^.

According to previous reports, some antimicrobial peptides can be activated by both Toll and IMD pathways, such as Defensin and Metchnikowin in Drosophilla^15,44^, In *P*. *vannamei*, RNAi-mediated knockdown of Akirin significantly reduced the expression of NF-*κ*B dependent *Lv*ALF-AA-K, *Lv*crustin-P and *Lv*Pen3 and the transcription factors, Dorsal and Relish post-*V*. *anguillarum* and *M*. *lysodeikticu* challenge. These findings suggested that ALF, crustin, and Pen3 might be regulated by both Toll and IMD pathways^54^. Our result together with others implied that Toll and IMD pathways could interact synergistically to induce the independent activation of overlapping target genes. For instance, some target promoters might contain TF-binding sites for both Dif and Relish. Therefore, both pathways could activate them independently, and the response depends on the affinity and number of NF-*κ*B sites^55^. Cooperation regulation is sometime mediated through an interaction of the NF-*κ*B-related transcription factors in the two pathways. As stated previously, ALF*Pm*3 was the most responsive ALF*Pm* isoform during pathogen infections^13^. Our results supported this finding based on the observation that Toll and IMD pathways caused independent activation of ALF*Pm*3 genes and, in turn, enhanced ALF*Pm*3 gene expression.

**Figure 6:**
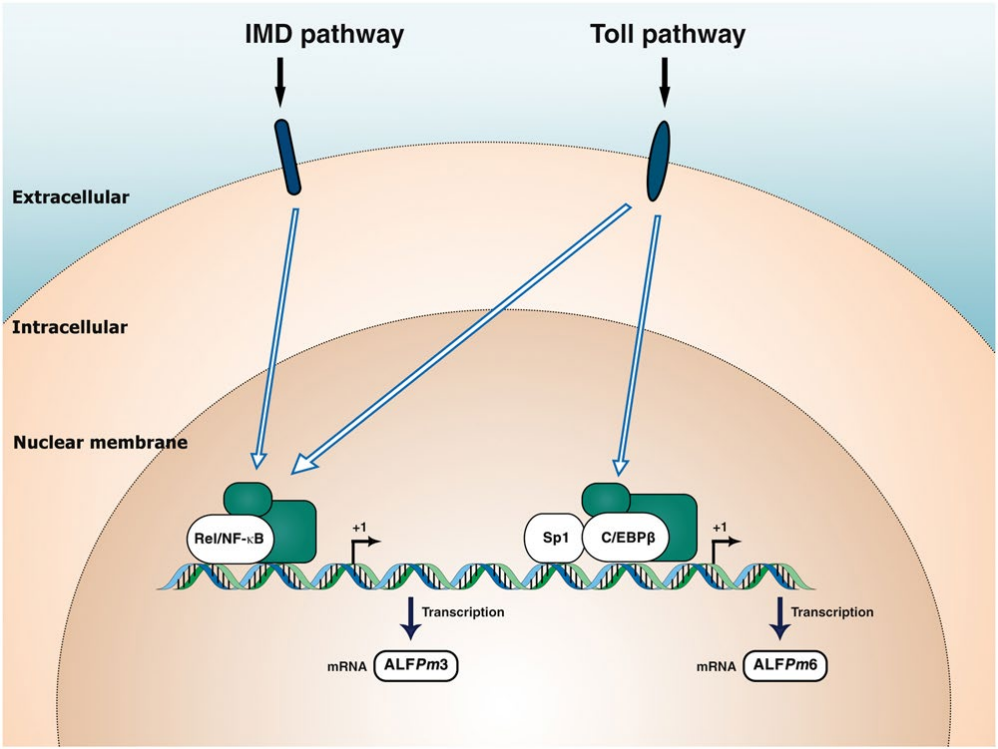
A schematic model of the signalling pathway involved in ALF*Pm*3 and ALF*Pm*6 transcriptional regulation.

Overall, in this study, we demonstrated the difference in gene regulation of ALF*Pm* against bacterial infection. ALF*Pm*3 gene expression was regulated by Toll and IMD pathways upon bacterial infection. Promoter analysis implied that ALF*Pm*3 transcription is positively modulated by the Rel/NF-*κ*B transcription factor. Furthermore, ALF*Pm*6 mRNA expression was regulated by the Toll pathway during bacterial challenge. C/EBP*β* and Sp1 transcription factors may be the activator that controlling the expression of ALF*Pm*6 (Fig. 6).

## Materials and Methods

### Peptide synthesis

The amino acid sequences (YCSFNVTPKFKRWQLYFRGRMWCP) corresponding to the cyclic LPS-BD of ALF*Pm*6 (accession number: AER45468) with a flanking amino acid residue at both the N and C terminal ends, in which a disulphide bond was formed between two cysteine residues, was designed and synthesized service by BIO BASIC INC., Canada. The lyophilized cALF*Pm*6#29–52 was resuspended in ultrapure-water and an aliquot was kept at −20 °C until used.

### Antimicrobial assay

The bacterial strains including the Gram-negative bacteria *E. coli* 363, *K. pneumoniae*, *E. cloacae*, *V. harveyi*, non-virulent *V. parahaemolyticus*, *V.* *parahaemolyticus* (AHPND) and *E. carotovora*, and the Gram-positive bacteria *B. megaterium*, *A. viridans*, *M. luteus*, *S. haemolyticus* and *S. aureus*, were used for the antibacterial activity assay.

The liquid growth inhibition assay was performed as previously described^24^ to test the antibacterial activity of cALF*Pm*6#29–52 peptide. Briefly, various concentrations of cALF*Pm*6#29–52 peptide (25 *μ*M to 100 *μ*M final concentration) were mixed with the mid-logarithmic growth phase of the bacterial culture in a 96-well plate. Bacteria were grown overnight under vigorous shaking at 30 °C or 37 °C according to the strains. By observation, a bacterial-peptide mixture with high turbidity is considered a the negative result. Only positive results with clear solution as a control were subjected for confirmation of the bacterial killing activity of cALF*Pm*6#29–52 peptide. A 20 *μ*l-aliquot of the bacterial culture incubated with the synthetic cALF*Pm*6#29–52 peptide was plated on an LB agar plate and incubated at 30 °C for 16 h. As a positive control, sterile water was used instead of the tested peptide. The number of bacterial colonies that grew on the LB agar plate was determined. The minimum bactericidal concentrations (MBCs) of the synthetic cALF*Pm*6#29–52 peptide was defined as the range between the highest concentration of the peptide where bacterial growth was observed and the lowest concentration of the peptide that killed the bacteria. To further investigate how the peptide inhibited the growth of bacteria, one drop of the above peptide-bacteria mixture was placed on a microscope slide with the bacterial sample and then Gram-stained and observed under a 100X magnification light microscope (Olympus CX31). Images were obtained using a microscope camera (Nikon DS-Fi1).

### Determination of the 5′-upstream sequences of the ALF*Pm*3 and ALF*Pm*6 genes

The 5′-upstream sequences of ALF*Pm*3 and ALF*Pm*6 genes were determined using the BD GenomeWalker Universal Kit (Clontech Laboratories, Inc., USA). The restriction enzyme-digested *P*. *monodon* genomic DNA libraries (E*coR*V, *Dra*I, *Pvu*II and *Stu*I) were used as template. The primary PCR using the outer adaptor primer (AP1) and the first gene-specific primer (GSP1) was amplified. The secondary PCR was performed with the nested adaptor primer (AP2) and a nested gene-specific primer (GSP2). The GSP1 and GSP2 primers designed from the known genomic sequence of ALF*Pm*3 (accession no. EF523562) and ALF*Pm*6 (accession no. JN562340) are shown in Supplementary Table S1. The nested PCR products were cloned into the pGEM-T easy vector (Promega, USA) and analysed by a DNA sequencing service (Macrogen Inc., Korea). The putative start sites and TATA box were predicted using the Promoter 2.0 Prediction Server (www.cbs.dtu.dk) and BDGP (Neural Network Promoter Prediction) (www.fruitfly.org). The putative cis-regulatory elements in the 5′-upstream sequences were predicted by TF search Version 1.3, Match 1.0 and the Alibaba 2 analysis programme (TRANSFAC® Public database) (www.gene-regulation.com). The results were compared with the JASPAR database (jasper.genereg.net) to confirm the putative transcription factor binding sites.

### Construction of ALF*Pm* promoter reporter plasmids

To construct the luciferase reporter plasmid harbouring various fragments of promoter sequences, the ALF*Pm*3 and ALF*Pm*6 promoters were randomly narrowed down. ALF*Pm*3 promoter fragments included the nucleotide positions of (−1478/+324), (−814/+324), (−719/+324), (−265/+324), and (−71/+324). For the ALF*Pm*6 promoter, various fragments at nucleotide positions (−419/+85), (−282/+85), (−162/+85), and (−80/+85) were constructed. Primer sequences containing the restriction sites *Nhe*I and *Bgl*II used for promoter fragment amplification are shown in Supplementary Table S1. The PCR cycle was initiated by denaturation at 95 °C for 1 min followed by 35 cycles of 95 °C for 30 sec, 60 °C for 30 sec, and 68 °C for 30 sec, and a final extension at 68 °C for 10 min. The products were purified using Nucleospin® Extract II kit (Macherey-Nagel) and cloned into the pGL3-basic vector (Promega). All recombinant plasmid constructs were purified and sequenced (Macrogen Inc., Korea).

### Promoter activity assay

*Drosophila Schneider* 2 (S2) cells were seeded in a 24-well plate and cultured in complete Schneider’s Drosophila Medium containing 10% heat-inactivated FBS and antibiotics (50 units penicillin G and 50 *μ*g/ml streptomycin sulphate, Invitrogen) at 27 °C for overnight. The 200 ng of pGL3-Basic (control plasmid) or pGL3 plasmid containing either ALF*Pm*3 or ALF*Pm*6 promoter fragments were co-transfected with 50 ng of the pRL-TK plasmid containing the Renilla luciferase gene into the S2 cells using Effectene® transfection reagent (Qiagen) according to manufacturer’s protocol. Cell lysates were collected at 48 h after transfection. Firefly luciferase and Renilla luciferase activities were assayed using the Dual-Luciferase® Reporter Assay System (Promega). Luminescence was detected using a SpectraMax M5 Multi-Mode Microplate Reader (Molecular device). The relative light unit (RLU) was calculated by normalizing the firefly luciferase activity to the Renilla luciferase activity to correct the transfection efficiency. The data were reported as the relative luciferase activity. Independent triplicate experiments were performed for each construct, and the standard deviation was calculated.

### Promoter deletion assay and site-directed mutagenesis

The obtained promoter active region was further analysed for the specific transcription factor binding site involved in ALF*Pm* gene regulation. To determine whether the 21 units of GAAAGAGAGTAAGAG[T/C] tandem repeat was involved in regulating ALF*Pm*3 gene expression, the tandem repeat deletion construct, del(−693/−358)/p(−1478/+302), was prepared by rolling PCR using p(−1478/+324) as a template and the primer pair del(−693/−358)/p(−1478/+324)F and del(−693/−358)/p(−1478/+324)R (Table S1). The PCR condition were 94 °C for 2 min, followed by 35 cycles of 98 °C for 10 s, 60 °C for 30 s, and 68 °C for 7 min, using KOD Taq polymerase (TOYOBO). A site-directed mutagenesis technique was performed to identify the putative binding site for the transcription factor that regulates ALF*Pm* genes. For ALF*Pm*3, the promoter transcription factor binding sites of Sp1 (−719/−701) and NF-*κ*B (−280/−270) were mutated using p(−1478/+324) as a template. For the ALF*Pm*6 promoter region, mutation of the transcription factor binding sites of Sp1 (−249/−238), ICSBP (−147/−136) and C/EBP*β* (−78/−88) were performed using p(−419/+85) as a template. The mutated constructs of these transcription factor-binding sites were prepared by rolling PCR. The conserved nucleotides of the interested TF-binding site were identified by JASPAR (an open-access database for eukaryotic TF-binding profiles) and their specific primers with mutated nucleotides were designed (Supplementary Table S1). The PCR conditions were 94 °C for 2 min, followed by 35 cycles of 98 °C for 10 s, 60 °C for 30 s, and 68 °C for 7 min, using KOD Taq polymerase (TOYOBO). For each construct, the PCR product was ligated and transformed into *E*. *coli* strain XL-1 blue by electroporation. The recominant plasmids were extracted using the High-speed plasmid mini kit (Geneaid). The mutated-transcription factor-binding sites were confirmed by DNA sequencing (Macrogen Inc., Korea). The promoter activity of the mutant was measured and compared with wild type using the Dual-Luciferase® Reporter Assay (Promega).

### Silencing efficiency of MyD88 dsRNA and Relish dsRNA

The dsRNA specific to MyD88, Relish and GFP were prepared according the T7 RiboMAX™ Express RNAi System (Promega) kit’s instructions. DNA template containing the T7 promoter sequence at the 5′-end was generated by PCR using the specific primer, T7-MyD88-F, MyD88-R, MyD88-F, T7-MyD88-R, T7-Relish-F, Relish-R, Relish-F, T7-Relish-R, and T7-GFP-F, GFP-R, GFP-F, T7-GFP-R (Supplementary Table S1) to produce sense and antisense RNA strands separately. The single-stranded RNA was annealed to generate dsRNA. After purification, the dsRNA was quantified and stored at −80 °C until use.

For the dsRNA-mediated gene silencing experiments, 144 shrimps were randomly divided into four groups (n = 36 in each group). The experimental group (3–5 g shrimp) was treated with MyD88 dsRNA or Relish dsRNA (10 *μ*g/g shrimp), while the control groups were injected with GFP dsRNA and 0.85% NaCl, respectively. To test the silencing efficiency, the haemolymph of 9 individuals from each treatment was collected at 0, 24, 48, and 72 h post-dsRNA injection (hpi), and total RNA was extracted. Three sets of pooled samples were prepared, each comprising 3 different individuals. The first-strand cDNA was synthesized from 1 *μ*g of total RNA using the RevertAidTM First Strand cDNA Synthesis Kit (Thermo Scientific). Either MyD88 or Relish gene expression was analysed by quantitative real-time RT-PCR using the gene-specific primer pair, PmMyD88-RT_F, PmMyD88-RT_R, PmRelish-RT_F and PmRelish-RT_F, respectively (Supplementary Table S1). The elongation factor-1*α* (EF1*α*) gene was used as an internal control. The relative expression of MyD88 and Relish was calculated using a comparative method described by Pfaffl^56^. The data are shown as the means ± standard deviations. Statistical analysis was performed using the one-way ANOVA followed by Duncan’s new multiple range test. Data differences were considered statistically significant at *P* < 0.01.

### MyD88 dsRNA and Relish dsRNA-mediated gene knockdown in *V*. *harveyi*-infected shrimp

To investigate the effect of MyD88 and Relish gene knockdown on the ALF*Pm*3 and ALF*Pm*6 transcript levels upon *V*. *harveyi* infection, shrimp were injected with 10 *μ*g/g shrimp of the MyD88 dsRNA, Relish dsRNA or GFP dsRNA. After the first dsRNA injection for 12 h, shrimp were challenged with 10^6^  CFU/ml of *V*. *harveyi* mixed with 10 *μ*g/g shrimp dsRNA. At 0, 6 and 24 h post-*V*. *harveyi* infection, shrimp haemolymph was collected and used for total RNA extraction and cDNA synthesis. Transcript levels of ALF*Pm*3 and ALF*Pm*6 genes in each sample were analysed by quantitative real-time RT-PCR using the specific primers, qRT-ALFPm3_F/R, qRT-ALFPm6_F/R, respectively. The relative expression of ALF*Pm*3 and ALF*Pm*6 was calculated using a comparative method described by Pfaffl^56^. The data are shown as the means ± standard deviations. The statistical analysis was performed using the one-way ANOVA followed by Duncan’s new multiple range test. Data differences were considered statistically significant at *P* < 0.01.

## Electronic supplementary material

Supplementary Table S1
