# Supplementary material for: Regulation of antilipopolysaccharide factors, ALFPm3 and ALFPm6, in Penaeus monodon
Source: Sci Rep. 2017 Oct 4;7:12694. doi: 10.1038/s41598-017-12137-5 (PMC5627258; doi:10.1038/s41598-017-12137-5)
Supplement: Supplementary file 1 — Supplementary Table S1 [file 41598_2017_12137_MOESM1_ESM.pdf]

Regulation of antilipopolysaccharide factors, ALF*Pm*3 and ALF*Pm*6, in *Penaeus monodon*

Pitchayanan Kamsaeng[1], Anchalee Tassanakajon[1], Kunlaya Somboonwiwat[1,*]

[1]Center of Excellence for Molecular Biology and Genomics of Shrimp, Department of Biochemistry, Faculty of Science, Chulalongkorn University, Phayathai Rd., Bangkok 10330, Thailand

* kunlaya.s@chula.ac.th

Supplementary Table S1. Summary of primers used to this study

Primer name	Sequence (5'→3')
**For genome walking**	
GSP1_ALF*Pm*3_primary	CTTGCACTCGTGGCCGAGAAGTTCAGTT
GSP2_ALF*Pm*3_nested	AGCACCAGGCTTACCAGCACGGACACA
GSP1_ALF*Pm*6_primary	AGTTTGTTCGCAATGGCTGGCACCAAT
GSP2_ALF*Pm*6_nested	CACCACCACCACGAGGATCATGCTGAA
**For luciferase construction**	
ALF*Pm*3promo+324BglII_R	AGGCGCAGATCTCTTCTGCAAGGGAAATAAAGATAAC
ALF*Pm*3promo-1478NheI_F	ATGAGCTAGCGCTGGTCCTGTGGTAAG
ALF*Pm*3promo-814NheI_F	AGTAGCTAGCGAAGCAGAGCCTCGCTAT
ALF*Pm*3promo-719NheI_F	ATCTGCTAGCTGAGGAAGAATGTGCGAGTG
ALF*Pm*3promo-265NheI_F	GCCTGCTAGCGGAAATACGCGTTGCTGT
ALF*Pm*3promo-71NheI_F	TACGGCTAGCATAGGCTCCTGGCAACT
ALF*Pm*6promo+85BglII_R	ACTGAGATCTGATGGACGTGAAGTGAAG
ALF*Pm*6promo-419NheI_F	TTACGCTAGCCCCGGGCTGGTAAATG
ALF*Pm*6promo-282NheI_F	ATCGGCTAGCGGCTGGTATTCCCAAGTCT
ALF*Pm*6promo-162NheI_F	AGCGGCTAGCACATGTATGAATGCCGAAAACG
ALF*Pm*6promo-80NheI_F	CAGCGCTAGCACATTTATGCACCCATCTCC
**For deletion assay and site-directed mutagenesis**	
del(-693/-358)/p(-1478/+324)F	AGAGAGGGGGGGGAGAGG
del(-693/-358)/p(-1478/+324)R	CTTTCACTCGCACATTCTTCCTCACTC
p(-814/+324)muNF-kB_F	CGTTGCTGTTACTATAGTTTCCACGTCG
p(-814/+324)muNF-kB_R	CGTATTTCCAGCTGAGAAAGATACTGCC
p(-282/+85)muICSBP_F	ACATGTATGAATGCCGCACACTCCAACTGAGCTG
p(-282/+85)muICSBP_R	ATATCTATACATAATGGACTTTGG
p(-282/+85)muC/EBPβ_F	AATGTATAAAGTAACATTTATGCACC
p(-282/+85)muC/EBPβ_R	ATTCACACATTATATGTTATTCTCA
**For dsRNA synthesis**	

**PmMyD88 dsRNA**	
Sense strand template	
T7-MyD88-F	TAATACGACTCACTATAGGG-CCTCAGCAAAGGTCTTGAAC
MyD88-R	CAGTCCACCAATTAGGTCTC
Anti-sense strand template	
MyD88-F	CCTCAGCAAAGGTCTTGAAC
T7-MyD88-R	TAATACGACTCACTATAGGG-CAGTCCACCAATTAGGTCTC
**PmRelish dsRNA**	
Sense strand template	
T7-Relish-F	TAATACGACTCACTATAGGG-CTCGTGGTCAGGAAGACTCAAT
Relish-R	GACTGGAGATGGAGACTGAATG
Anti-sense strand template	
Relish-F	CTCGTGGTCAGGAAGACTCAAT
T7-Relish-R	TAATACGACTCACTATAGGG-GACTGGAGATGGAGACTGAATG
**GFP dsRNA**	
Sense strand template	
T7-GFP-F	TAATACGACTCACTATAGGG-ATGGTGAGCAAGGGCGAGGA
GFP-R	TTACTTGTACAGCTCGTCCA
Anti-sense strand template	
GFP-F	ATGGTGAGCAAGGGCGAGGA
T7-GFP-R	TAATACGACTCACTATAGGG-TTACTTGTACAGCTCGTCCA
**For RT-PCR**	
EF1α -F	GGTGCTGGACAAGCTGAAGGC
EF1α -R	CGTTCCGGTGATCATGTTCTTGATG
PmMyD88-RT_F	GTGCACCAGAGTCATTGTAG

PmMyD88-RT_R	GGGAGTGGCAGAAACTTATC
PmRelish-RT_F	TCTCCAGGTGAGCACTCAGTTG
PmRelish-RT_R	GCTGTAGCTGTTGCTGTTGTTGAG
**For quantitative realtime RT-PCR**	
qRT-ALF*Pm*3_F	CCCACAGTGCCAGGCTCAA
qRT-ALF*Pm*3_R	TGCTGGCTTCTCCTCTGATG
qRT-ALF*Pm*6_F	AGTCAGCGTTTAGAGAGGTT
qRT-ALF*Pm*6_R	GCTCGAACTCTCCACTCTC
